# Activation of the Connective Tissue Growth Factor (CTGF)-Transforming Growth Factor β 1 (TGF-β 1) Axis in Hepatitis C Virus-Expressing Hepatocytes

**DOI:** 10.1371/journal.pone.0046526

**Published:** 2012-10-04

**Authors:** Tirumuru Nagaraja, Li Chen, Anuradha Balasubramanian, Jerome E. Groopman, Kalpana Ghoshal, Samson T. Jacob, Andrew Leask, David R. Brigstock, Appakkudal R. Anand, Ramesh K. Ganju

**Affiliations:** 1 Department of Pathology, Ohio State University Wexner Medical Center, Columbus, Ohio, United States of America; 2 Center for Clinical and Translational Research, The Research Institute at Nationwide Children's Hospital, Columbus, Ohio, United States of America; 3 Division of Experimental Medicine, Beth Israel Deaconess Medical Center, Harvard Medical School, Boston, Massachusetts, United States of America; 4 Department of Molecular and Cellular Biochemistry, The Ohio State University, Columbus, Ohio, United States of America; 5 Schulich School of Medicine and Dentistry, University of Western Ontario, London, Ontario, Canada; University of South Carolina School of Medicine, United States of America

## Abstract

**Background:**

The pro-fibrogenic cytokine connective tissue growth factor (CTGF) plays an important role in the development and progression of fibrosis in many organ systems, including liver. However, its role in the pathogenesis of hepatitis C virus (HCV)-induced liver fibrosis remains unclear.

**Methods:**

In the present study, we assessed CTGF expression in HCV-infected hepatocytes using replicon cells containing full-length HCV genotype 1 and the infectious HCV clone JFH1 (HCV genotype 2) by real-time PCR, Western blot analysis and confocal microscopy. We evaluated transforming growth factor β1 (TGF-β1) as a key upstream mediator of CTGF production using neutralizing antibodies and shRNAs. We also determined the signaling molecules involved in CTGF production using various immunological techniques.

**Results:**

We demonstrated an enhanced expression of CTGF in two independent models of HCV infection. We also demonstrated that HCV induced CTGF expression in a TGF-β1-dependent manner. Further dissection of the molecular mechanisms revealed that CTGF production was mediated through sequential activation of MAPkinase and Smad-dependent pathways. Finally, to determine whether CTGF regulates fibrosis, we showed that shRNA-mediated knock-down of CTGF resulted in reduced expression of fibrotic markers in HCV replicon cells.

**Conclusion:**

Our studies demonstrate a central role for CTGF expression in HCV-induced liver fibrosis and highlight the potential value of developing CTGF-based anti-fibrotic therapies to counter HCV-induced liver damage.

## Introduction

Chronic hepatitis C virus (HCV) infection is a leading cause of end-stage liver disease, including liver cirrhosis and hepatocellular carcinoma, with approximately 3% of the world's population infected (130–170 million individuals) [1]. The main targets of HCV infection are human hepatocytes, where HCV not only causes an inflammatory response, but also activates pro-fibrogenic pathways that contribute to liver fibrosis [2]. Liver fibrosis is characterized by the production of pro-fibrogenic cytokines by parenchymal cells (hepatocytes) and mesenchymal cells e.g. Kupffer cells, endothelial cells, hepatic stellate cells (HSCs), which collectively contribute to the unrelenting synthesis and deposition of extracellular matrix (ECM) components, downregulation of matrix metalloproteinases (MMPs) and increased expression/action of tissue inhibitor of metalloproteinases (TIMPs) [2], [3]. Together, these molecular changes determine the progression of chronic hepatitis C to liver cirrhosis and hepatocellular carcinoma (HCC) [1].

Recently, the profibrogenic cytokine connective tissue growth factor (CTGF), a member of the CCN gene family (CTGF, cyr61/cef10, nov), has been shown to play a key role in various fibrotic disorders [3], [4], [5], [6], [7]. It is a multi-functional protein (∼40 kD) produced by various cell types that acts via autocrine or paracrine pathways to regulate diverse cellular functions including growth, proliferation, apoptosis, adhesion, migration, ECM production and differentiation [8]. The receptors for CTGF on various cells have, however, not been well-characterized [9]. Data reported in recent years provides compelling evidence that CTGF is a key factor in development of hepatic fibrosis [3], [10], [11], [12], [13], [14]. With regard to HCV infection, CTGF expression in liver biopsy samples has been shown to correlate independently with the fibrosis stage and plasma HCV RNA levels [11], [15]. In the present study, we investigated the role of CTGF in HCV-induced liver fibrosis and the molecular mechanism of its production.

The fibrogenic mechanisms in the liver are dependent on the interplay of many pro- and anti-fibrotic cytokines. CTGF is often co-expressed with transforming growth factor β1 (TGF-β1) in various fibrotic disorders. TGF-β1 is a key profibrogenic cytokine in the liver, participating in many critical events leading to liver fibrosis, such as HSC activation, hepatocyte apoptosis, ECM formation and expression of other profibrogenic mediators. Furthermore, TGF- β1 has also been shown to facilitate epithelial-to-mesenchymal transition of hepatocytes that in turn participates in the progression of liver fibrosis [16], [17], [18]. Clinical studies have revealed elevated TGF-β1 serum levels in patients with chronic hepatitis B virus (HBV)/HCV infections [19], [20]. Studies in several connective tissue cell types have shown that CTGF acts as a potent downstream mediator of TGF-β1, modulating its functional effects [10]. However, the cross-talk between these profibrogenic cytokines during HCV infection is not known. In the present study, we first demonstrated the upregulation of CTGF and TGF-β1 in the well-characterized Huh7.5-FL HCV replicon system and HepG2 cells transfected with HCV JFH1 RNA. We further investigated the inter-relationship between TGF-β1 and CTGF in HCV infection. Our studies reveal that HCV-stimulated CTGF is induced downstream of TGF-β1 in a MAPKinase and Smad-dependent manner and that CTGF production drives production of key fibrosis-associated markers, including procollagen I. The central role of CTGF production in HCV-infected hepatocytes highlights the potential value of developing CTGF-based anti-fibrotic therapies to counter HCV-induced liver damage.

## Materials and Methods

### Antibodies

The antibodies used in the study were HCV NS5B (Alexis Biochemicals, San Diego, CA), HCV Core (Abcam, Cambridge, MA), HCV NS4A, TGF-β1 (Chemicon, Temecula, CA), Phospho-Smad2, Phospho-Smad3, Smad2, Smad3, Phospho-P38, P-38, Phospho-JNK, JNK, vimentin and Slug (Cell Signaling, Danvers, MA), TGF-β receptor I, Phospho-ERK, ERK, CTGF, Procollagen I and GAPDH (Santa Cruz Biotechnology, Santa Cruz, CA) and α-SMA (Sigma, St. Louis, MO).

### Cell cultures

In this study we used HCV- negative human hepatoma cell line Huh7.5 cells and Huh7.5 cells harboring full genome length HCV [Con1/FL-Neo HCV1b FL (S2204I)] (Huh7.5-FL) replicon cells (Apath, LLC; St. Louis, MO) and propagated in complete Dulbecco's modified Eagle's medium (DMEM) (Invitrogen, Carlsbad, CA). Huh 7.5 cells represent a Huh7 subline which are cured with interferon to render them highly permissive to HCV replication [21]. Huh7.5-FL cells were maintained in medium containing 750 µg/ml of Geneticin (G418) [21]. The HepG2 cell line was grown in complete Eagle's minimal essential medium and used for transfection with HCV genotype 2A (JFH1; Japanese fulminant hepatitis) RNA.

### Western Blot analysis

Equivalent amounts of protein extracts were run on a 4–12% gradient polyacrylamide gel (Invitrogen). Separated proteins were transferred to nitrocellulose membranes, which were probed with specific antibodies and developed using the enhanced chemiluminescence detection system (GE Healthcare, Piscataway, NJ).

### Quantitative reverse-transcriptase polymerase chain reaction (RT-PCR)

RNA extraction and real-time PCR for CTGF and TGF-β1 were performed as described before [22]. Briefly, total RNA was extracted from the Huh 7.5 or Huh7.5-FL cells, first-strand cDNA synthesized and real-time PCR reactions performed using SYBR Green Master Mix kit (Applied Biosystems, Forster City, CA). The sequences of the primers used were: CTGF forward primer: AATGCTGCGAGGAGTGGGT; CTGF reverse primer: CGGCTCTAATC ATAGTTGGGTCT; TGF-β1 forward primer: ACCTGAACCCGTGTTGCTCT; TGF-β1 reverse primer: CTAAGGCGAAA GCCCTCAAT; GAPDH forward primer: TGCACCACCAACTGCTTAGC; GAPDH reverse primer: GGCATGGACTGTGGTCATGAG; TGF-β RI forward primer ATTACCTGGACATCGGCA AC; TGF-β RI reverse primer TTGGGCACCACATCATAGAA (Operon Biotechnology, Huntsville, AL). Negative controls were a non-reverse transcriptase reaction or a non-sample reaction. GAPDH was amplified as an internal standard.

### ELISA assays

Conditioned medium (DMEM containing 0.5% FCS) from Huh 7.5 or Huh7.5-FL cells were collected at different time points, centrifuged and the supernatant used for determination of biologically active TGF-β1 protein by ELISA (BD Biosciences) according to the manufacturer's instructions.

### Immunofluorescence

Fixed cells were stained with primary antibodies including NH1 anti-CTGF IgY (5 µg/ml) [23], HCV NS5B, NS4A; Core or anti-TGF-β1 (1∶200, Santa Cruz) followed by incubation with secondary antibodies Alexa Fluor® 568 goat-anti chicken IgY (1∶1000) or Alexa Fluor® 568 goat-anti rabbit IgG (1∶400) or Alexa Fluor® 488 goat-anti mouse IgG (1∶400) (Invitrogen). The cells were mounted with Vectashield Mounting Medium containing DAPI (Vector Laboratories, Burlingame, CA), and examined by confocal laser microscopy (LSM510, Carl Zeiss, Jena, Germany).

### Transfections and DNA Constructs

To transfect HepG2 cells with full length JFH1 RNA, pJFH1-pUC plasmid (Apath, LLC) as a DNA template and MEGAscript T7 RNA synthesis kit (Applied Biosystems) were used. 5 µg of RNA was transfected into HepG2 cells using Nucleofector V solution in an Amaxa nucleofector device (program no. T-028). Cell lysates were collected at various time points and used for the analysis of multiple proteins by Western blot analysis.

To determine Smad-dependent CTGF promoter activity, Huh7.5 and Huh7.5-FL cells were transfected with plasmids containing a secreted alkaline phosphatase (SEAP) reporter gene fused to either the wild type CTGF promoter (nucleotides −805 to +17) or individual point mutants targeting the Smad site or the basal control element (BCE-1) using Lipofectamine ™ 2000 (Invitrogen). Promoter/reporter constructs contained CCN2 promoter fragments spanning nucleotides −805 to +17 (wild type promoter) and mutations in the Smad element (TCAGA to GGATC) and GGAA element (GGAAT to TCCCG) introduced into the CCN2 promoter between nucleotides −805 to +17, but were otherwise identical to construct −805.

SEAP reporter activity was calculated after adjustment for differences among samples in transfection efficiency as determined by co-transfection with a cytomegalovirus (CMV) promoter-β-galactosidase (CMV-β-gal) reporter gene. CTGF-SEAP promoter activity assays were performed with Phospha-Light kit and β-galactosidase expression was determined by Galacto-star kit (Applied Biosystems). SEAP levels were measured using an LMax II 384 luminometer (Molecular Devices, Sunnyvale, CA).

### ShRNA-mediated suppression of TGF-β1 or CTGF

Human TGF-β1 5′-ACGAGC CCTGG ACACCAACTAT-3′(sense) and 3′- ATAGTTGG TGTCCAGGGCTCGG 5′ (antisense) or CTGF 5′-CCAGCACCAGAATGTATATTAA-3′(sense) and 3′- TTAATATACATTCTGGTG CTGT-5′ (antisense) GIPZ lentiviral shRNA plasmids or negative scrambled shRNA (Open Biosystems, Huntsville, AL) were transfected into Huh7.5 or Huh7.5-FL cells using Lipofectamine ™ 2000 according to the manufacturer's instructions. Transfection efficiency was monitored by co-transfection with the green fluorescent protein (GFP)-expressing plasmid, pEGFP (Invitrogen).

### Cultivation of Human hepatic stellate cells (LX2 cells) with conditioned medium from Huh7.5 and Huh7.5-FL cells

To analyze the role of profibrogenic cytokines in the supernatants of Huh7.5 and Huh7.5-FL on HSCs, we incubated hepatic stellate cells (LX2) with the respective supernatants for 48 hrs, after which the HSCs were lysed and analyzed for procollagen I. LX2 cells were kindly provided by Dr. Scott Friedman (Mount Sinai School of Medicine, New York).

### Statistical analysis

All the experiments were carried out in triplicate or quadruplicate. Each set of experiments was repeated at least three times with similar results in each case. Representative graphical data are presented as mean ± standard deviation. Student's t test for paired samples was used to determine statistical significance. Differences were considered significant at p≤0.05.

## Results

### Demonstration of active HCV replication in the HCV culture system

**Figure 1:**
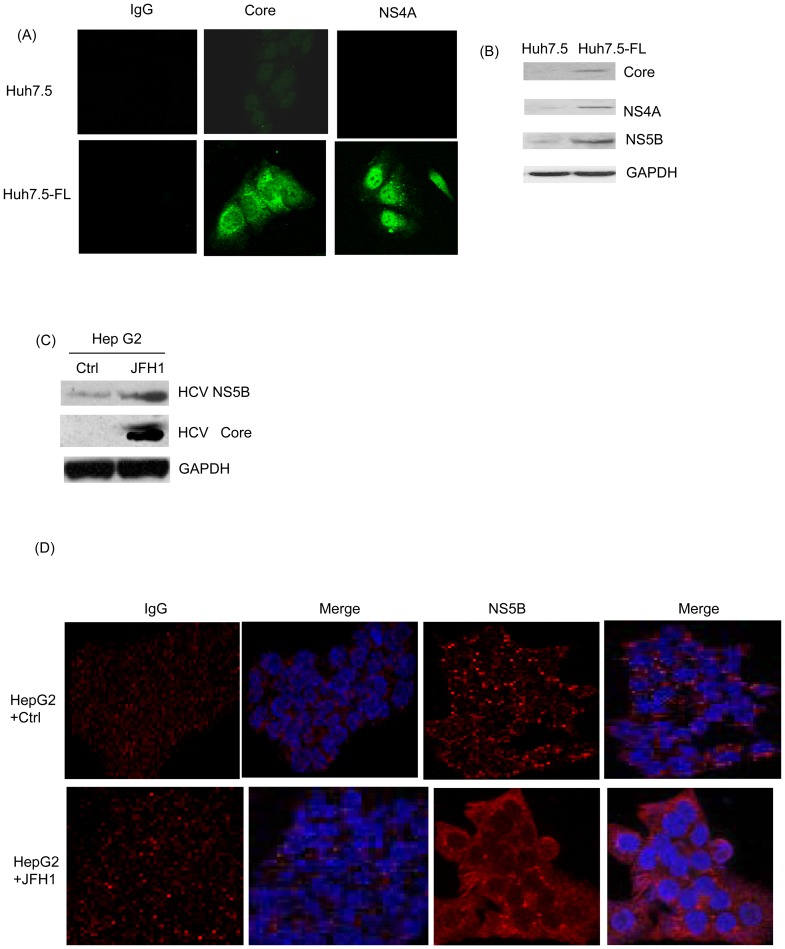
Detection of active HCV replication in HCV-infected cell lines. (**A**) Huh7.5 or Huh7.5-FL cells grown for 48 h were stained with antibodies against HCV proteins (HCV core or NS4A), followed by FITC-coupled secondary antibodies and detected using confocal microscopy. (**B**) Lysates of cells grown for 48 hours were analyzed for expression of HCV core, NS4A or NS5B by Western blotting. HepG2 cells were transfected with JFH-1 RNA and expression of HCV NS5B and core protein was analyzed by Western blotting (**C**) and confocal microscopy respectively (**D**). Equal protein loading in the Western blot analyses was verified using GAPDH antibody. Data are from one of three independent experiments performed in triplicate.

Firstly, we validated the presence of active HCV at the protein level in Huh7.5-FL cells stably expressing genome-length HCV. The expression of core and NS4A protein in Huh7.5-FL cells was shown by confocal microscopy (Fig. 1A) and Western blot analysis (Fig. 1B). We also validated the findings with another HCV culture system by transfecting the JFH-1 (HCV genotype-2) RNA into the HepG2 cells. The expression of HCV NS5B and core protein in HepG2 cells was confirmed by Western blot (Fig. 1C) and confocal microscopy (Fig. 1D).

### CTGF expression and secretion is increased in HCV-infected hepatocytes

**Figure 2:**
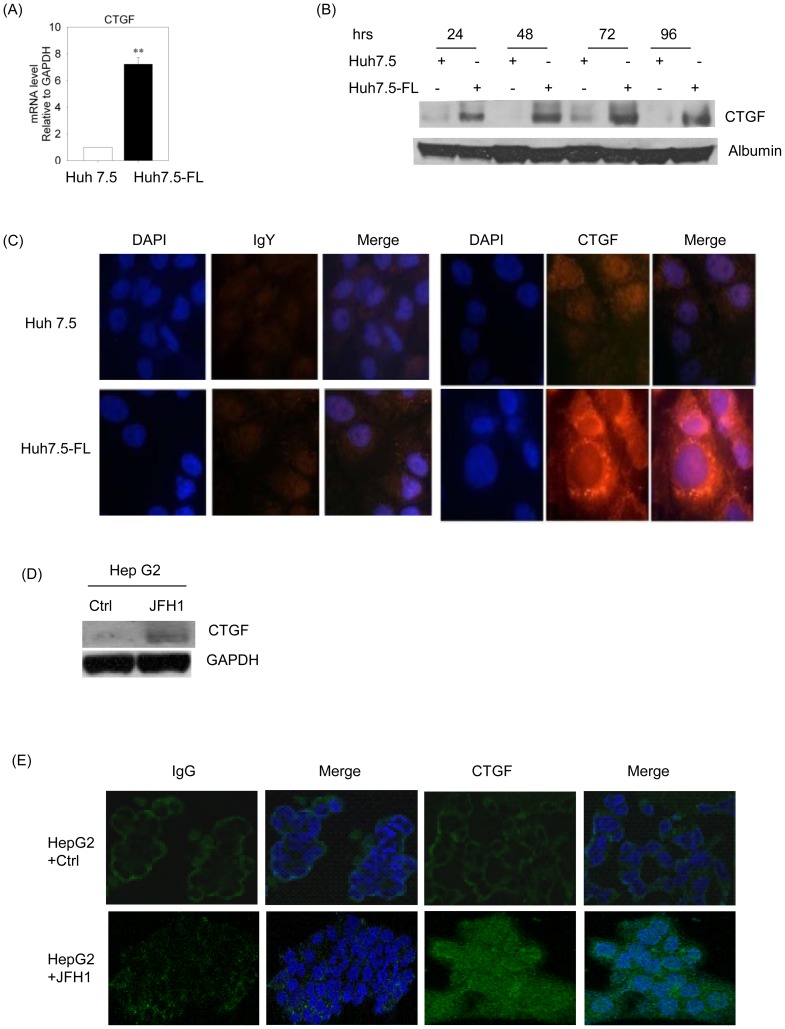
HCV induces CTGF expression in Huh7.5-FL cells. (**A**) RNA from Huh7.5 or Huh7.5-FL cells was used in the SYBR green real-time PCR to analyze CTGF expression. ** P<0.001 versus Huh7.5 cells. (**B**) Conditioned medium from Huh7.5 or Huh7.5-FL cells incubated for various time periods was collected, concentrated and equal amounts of protein subjected to SDS-PAGE and analyzed for CTGF by Western blotting. Albumin was used as a internal control. (**C**) Huh7.5 or Huh7.5-FL cells were grown for 48 hours, after which the cells were fixed, permeabilized and treated with anti-CTGF followed by FITC-coupled secondary antibodies and examined using an Olympus FV1000 confocal microscope. HepG2 cells were transfected with JFH1 RNA and CTGF expression was analyzed by (**D**) Western blotting and (**E**) confocal microscopy respectively. Equal protein loading was verified using antibodies against GAPDH. Data represent mean ± SD of 3 independent experiments.

We first evaluated the expression of CTGF in Huh7.5 or Huh7.5-FL cells after incubation in conditioned medium (DMEM containing 0.5% FCS). After 32 hours, Huh7.5-FL cells showed a 7-fold higher expression of CTGF mRNA levels by quantitative real time-PCR (RT-PCR) in comparison to the Huh7.5 cells (Fig. 2A). The presence of a 38 kDa CTGF band in the medium was significantly higher in Huh7.5-FL cells versus Huh7.5 cells as assessed by Western blot analysis (Fig. 2B). In addition, we demonstrated increased levels of cellular CTGF protein in Huh7.5-FL cells at 48 hours by confocal microscopy (Fig. 2C). Furthermore, we also used HepG2 cells transfected with the HCV genotype 2 (JFH1) RNA to evaluate CTGF expression. Western blot analysis of cell lysates and confocal microscopy of immuno-stained cells indicated that CTGF production was enhanced in JFH1-expressing cells, as compared to the control cells (Fig. 2D and E), thus verifying that CTGF production was stimulated in hepatocytes expressing HCV.

### CTGF mediates HCV-induced expression of fibrotic markers

**Figure 3:**
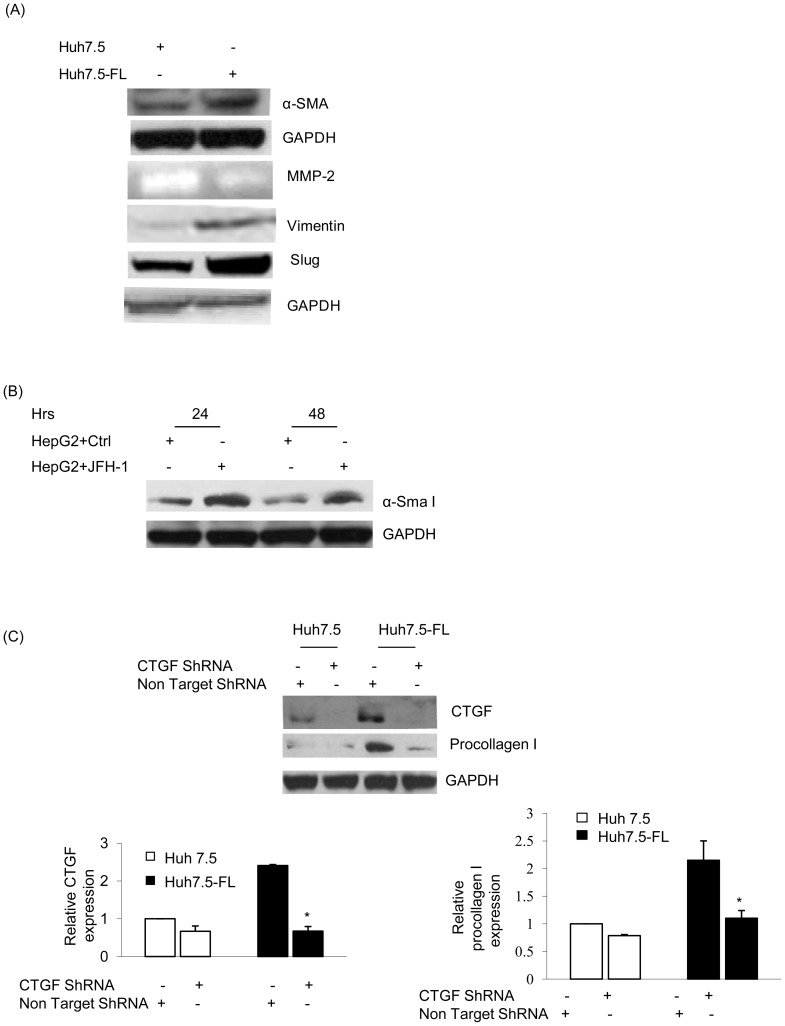
CTGF stimulates the expression of fibrotic markers in Huh7.5-FL cells. (**A**) Huh7.5 or Huh7.5-FL cells were incubated in conditioned medium (medium containing 0.5%FCS) for ninety-six hours and the cell lysates were blotted to examine α-SMA expression, vimentin and slug expression. Equal protein loading was verified using GAPDH antibody. The conditioned medium was used for the measurement of MMP-2 activity by zymography assay. (**B**) HepG2 cells were transfected with or without JFH-1RNA for different time points and cell lysates were blotted for α-Sma I protein. GAPDH was used as an internal control. (**C**) Lysates of Huh7.5 or Huh7.5-FL cells transfected with non targeting or CTGF shRNA for 48 hrs were blotted for CTGF, procollagen I or GAPDH. The bar graphs show the quantitative analysis of CTGF or procollagen I expression relative to that of GAPDH. * P≤0.05 versus Huh7.5-FL cells. Data represent mean ± SD of 3 independent experiments.

In the present study, we evaluated the expression of α-smooth muscle antigen (α-SMA), matrix-metalloprotease-2 (MMP-2), vimentin and slug in Huh7.5-FL cells. As compared to Huh7.5 cells, the Huh7.5-FL cells expressed higher levels of α-SMA, vimentin and slug, and showed reduced levels of MMP-2 activity (50% reduction) (Fig. 3A) at the end of the 96-hour culture period. We also evaluated the expression of α-SMA in HepG2 cells transfected with JFH1 RNA and found increased levels of α-SMA in comparison to the controls (Fig. 3B). As shown in Figure 3C, procollagen I was upregulated in Huh7.5-FL cells, but this was abrogated in cells transfected with CTGF shRNA plasmid, as compared to the scrambled shRNA transfected cells, demonstrating that CTGF directly mediates the production of procollagen.

### HCV-induced CTGF expression is TGF-β1 dependent

**Figure 4:**
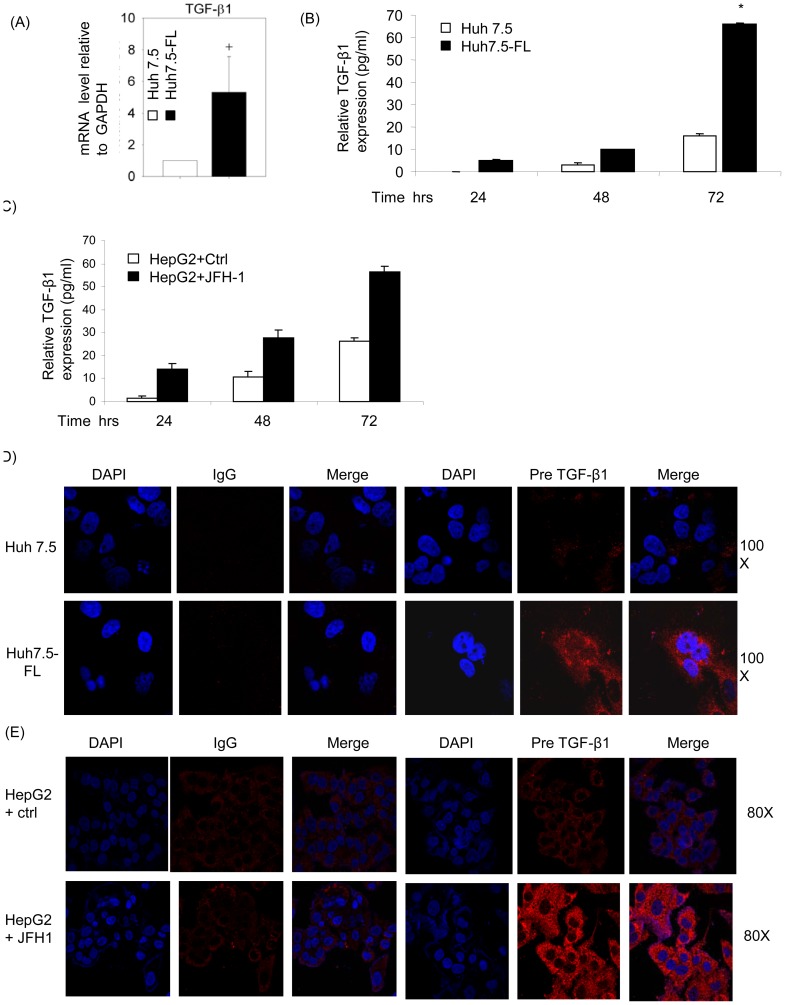
HCV induces TGF-β1 expression in Huh7.5-FL cells. (**A**) RNA was extracted from Huh7.5 or Huh7.5-FL cells which were incubated in conditioned medium for 32 hrs were used in SYBR green real-time PCR to analyze TGF-β1. + P<0.05 versus Huh7.5 control. (**B**) Huh7.5 or Huh7.5-FL cells were incubated for various time points and active TGF-β1 was measured in the culture supernatants by ELISA. * P≤0.05 versus Huh7.5 cells. (**C**) HepG2 cells were transfected with or without JFH-1 RNA and supernatants were collected at different time points to measure the TGF-β1 concentration in supernatant by ELISA. (**D**) Huh7.5 or Huh7.5-FL cells were fixed, permeabilized and stained with TGF-β1 antibody followed by FITC- coupled secondary antibody, and examined by confocal microscopy. (**E**) HepG2 cells transfected with or without JFH1 RNA were analyzed for TGF-β1 expression by confocal microscopy. Data are from one of three independent experiments performed in triplicate.

Several studies have shown that TGF-β1 is an important mediator of CTGF expression in various cell types [10], [24]. Hence, we evaluated the role of TGF-β1 in CTGF production by determining TGF-β1 mRNA and protein expression in Huh7.5 or Huh7.5-FL cells after various periods of incubation in conditioned medium. As compared to Huh7.5 cells, TGF-β1 mRNA was enhanced approximately 4-fold in Huh7.5-FL cells as assessed by quantitative RT-PCR (Fig. 4A). Furthermore, substantially higher amounts of active TGF-β1 were present in the conditioned media from Huh7.5-FL cells as compared to Huh7.5 cells at 24, 48 and 72 hours of culture in conditioned medium (Fig. 4B). Similarly, supernatant from HepG2 cells transfected with JFH1 RNA showed increased active TGF-β compared to control samples (Fig. 4C). We also confirmed the enhanced expression of TGF- β1 precursor in Huh7.5-FL versus Huh7.5 cells by confocal microscopy (Fig. 4D). In addition, we analyzed the expression of TGF-β1 precursor in HepG2 cells transfected with JFH1 RNA. Figure 4E shows the enhanced expression of TGF-β1 precursor in JFH1-transfected HepG2 cells by confocal microscopy.

**Figure 5:**
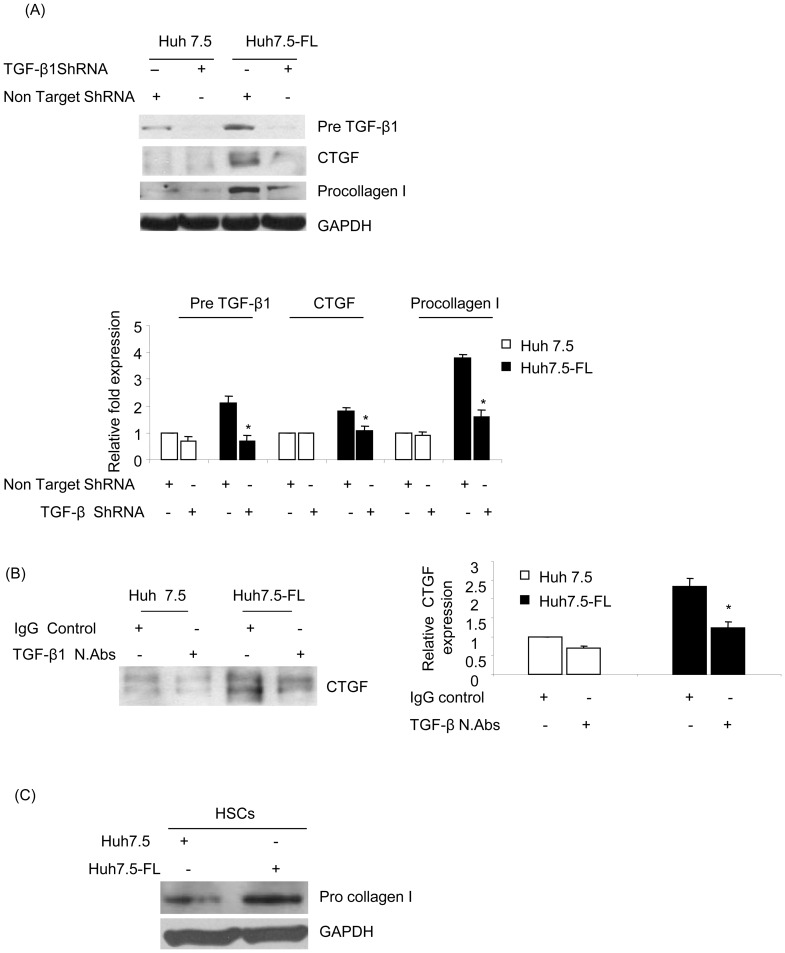
HCV-induced CTGF expression is TGF-β1-dependent. (**A**) Lysates of Huh7.5 or Huh7.5-FL cells transfected with non-targeting or TGF-β1 ShRNA for 48 hrs were blotted to determine expression of TGF-β1, CTGF or procollagen I (upper panel). Equal protein loading was determined using GAPDH antibody. The bar graph shows the quantitative analysis of TGF-β1, CTGF or procollagen I expression relative to that of GAPDH (lower panel). * P≤0.05 versus Huh7.5-FL cells. (**B**) Medium from Huh7.5 or Huh7.5-FL cells incubated for 48 hrs with anti-TGF-β1 or non-immune IgG was collected, concentrated and equal amounts of protein were used for Western blot analysis using CTGF antibody (upper panel). The bar graph shows the quantitative analysis of CTGF expression obtained by densitometry (lower panel). * P≤0.05 versus Huh7.5 cells. (**C**) Human hepatic stellate cells (LX2 cells) were co cultured with medium from Huh7.5 and Huh7.5-FL cells for 48 hrs and cell lysates were analyzed for procollagen I expression. GAPDH was used as an internal control. For all experiments, data represent mean ± SD of 3 independent experiments.

To establish the functional significance of TGF-β1 in CTGF production, Huh7.5 or Huh7.5-FL cells were transfected with either TGF-β1 or non-targeting shRNA plasmid. This treatment resulted in diminished TGF-β1 precursor protein levels in cell lysates, as expected, but also a concomitant decrease in CTGF secretion (Fig. 5A). Furthermore, treatment of the Huh7.5-FL cells with TGF-β1 neutralizing antibody resulted in highly diminished CTGF levels in conditioned medium (Fig. 5B). Taken together, these data indicated that elevated CTGF expression in HCV-infected hepatocytes is mediated through TGF-β1. As shown in Figure 5A (third panel), upregulation of procollagen I was abrogated in Huh7.5-FL cells transfected with TGF-β1 shRNA when compared with Huh7.5-FL cells transfected with scrambled shRNA. We further analyzed the role of profibrogenic cytokines on fibrotic marker expression in HSCs. Conditioned medium from Huh7.5 and Huh7.5FL cells were cocultured along with LX2 (Hepatic stellate cell line). As shown in figure 5C, we observed nearly two fold increase of procollagen I in LX2 cells incubated with medium from Huh7.5-FL cells compared to LX2 cells incubated with medium from Huh 7.5 cells.

### Signaling pathways that mediate HCV-induced CTGF-production via TGF-β1

**Figure 6:**
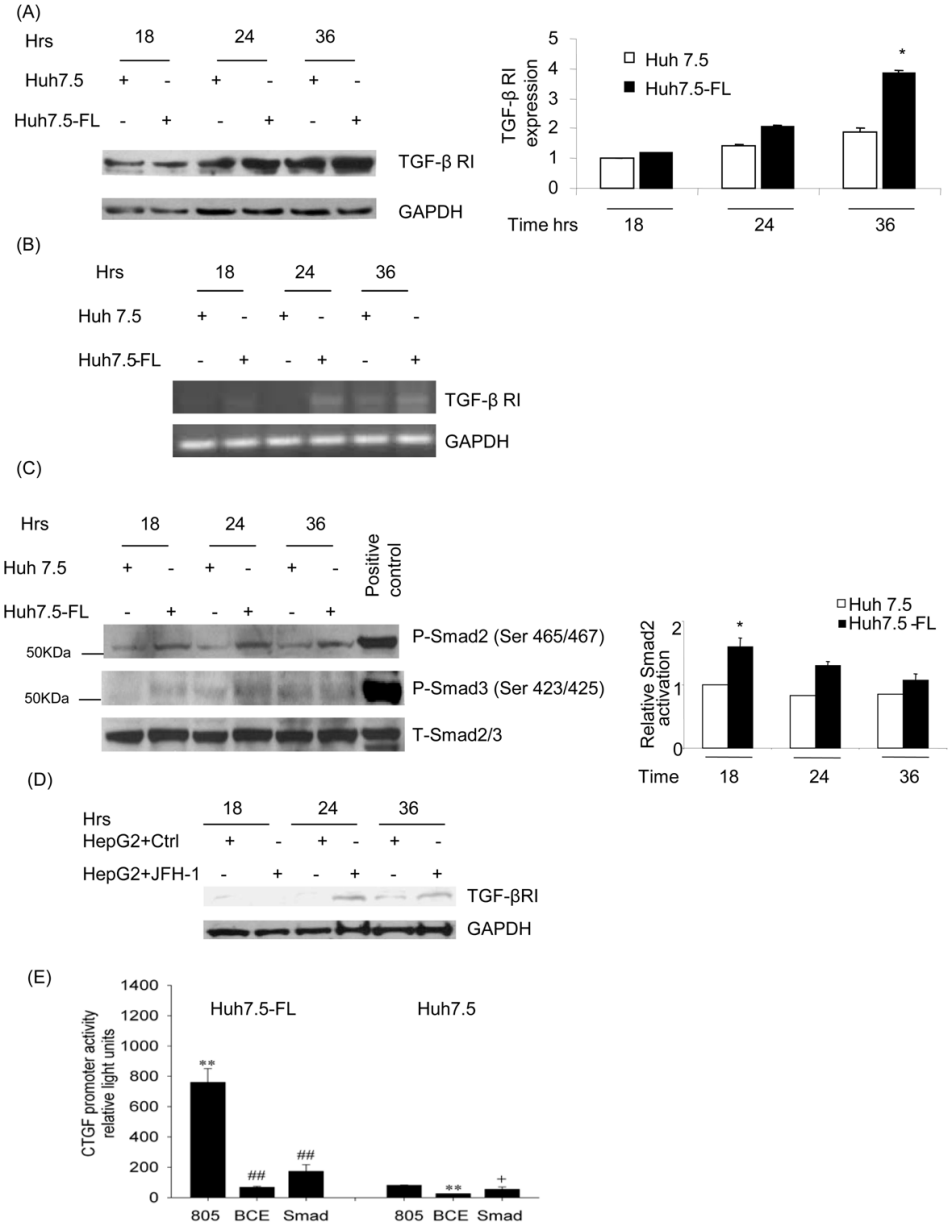
Expression of CTGF in Huh7.5-FL cells is Smad-dependent. Cell lysates from Huh7.5 or Huh7.5-FL cells were collected at different time points and blotted with anti-TGF-βRI (**A**) or phospho-Smad 2, phospho-Smad3 and total Smad2/3 antibodies (**C**). The bar graphs show the quantitative analyses of TGF-β RI and p-smad2 protein expression as obtained by densitometry. RNA from Huh7.5 or Huh7.5-FL cells was used in the reverse transcriptase PCR to analyze the TGF-β RI expression (**B**). (**D**) HepG2 cells were transfected with and without JFH-1 RNA. The cell lysates were collected at different time points and blotted for TGF-βRI. (**E**) Huh7.5 and Huh7.5-FL cells were transfected with different CTGF promoter/SEAP reporter constructs for 48 hrs. CTGF promoter activity was determined by measuring SEAP reporter expression. ** P<0.001 versus Huh7.5 cells; + P<0.05 versus Huh7.5 cells; and ## P<0.001 versus Huh7.5-FL cells. Data represent mean ± SD of 3 independent experiments.

We further explored the HCV-induced signaling mechanisms that mediate CTGF expression downstream of TGF-β1 in Huh7.5-FL cells. Previous studies have shown that TGF-β1 mediates its functional effects by binding to the TGF-β1-receptor complex and activating the Smad-dependent pathway. In the present study, we demonstrated the increased expression of TGF-β1 receptor I (TGFβR1/ALK5) in Huh7.5-FL cells and also in HepG2 cells transfected with JFH-1 RNA over the first 36 hours of culture by Western blot (Fig. 6A & 6D) and RT-PCR (Fig. 6B). To assess whether this difference was reflected in downstream signaling events, we evaluated phosphorylation of Smad2 and Smad3 in Huh7.5 and Huh7.5-FL cells. We observed a significant increase in Smad2 and Smad3 phosphorylation in Huh7.5FL cells compared to Huh 7.5 cells (Fig. 6C) after various time points of incubation in conditioned medium. Previous studies in other cell types showed that TGF-β-mediated induction of CTGF mRNA relies on the functional Smad element in the CTGF promoter and that, while the BCE-1 site is involved with basal CTGF promoter activity, it is also indirectly responsive to TGF-β since it is a response element for endothelin 1 which is induced by TGF-β and is essential for TGF-β to induce CTGF [25], [26].

Next, to determine the elements in the CTGF promoter involved downstream of HCV-induced TGF-β1, we transfected Huh7.5 or Huh7.5-FL cells with CTGF promoter reporters that were either wild-type (805) or that contained point mutations in either the BCE-1 (a response element that is indirectly regulated by TGF-β1) or the Smad binding site (which is directly controlled by TGF-β1). First, we found that the level of wild-type CTGF promoter activity in lysates from the Huh7.5-FL cells was approximately 10-fold higher than in those from Huh7.5 cells (Fig. 6E), consistent with earlier data showing enhanced CTGF mRNA and protein production in the Huh7.5-FL cells (Fig. 2). Secondly, the mutant promoter activities were substantially attenuated, an effect that was particularly evident in the Huh7.5-FL cells, resulting in reduction in activity of 95% or 90% respectively (Fig. 6E).

**Figure 7:**
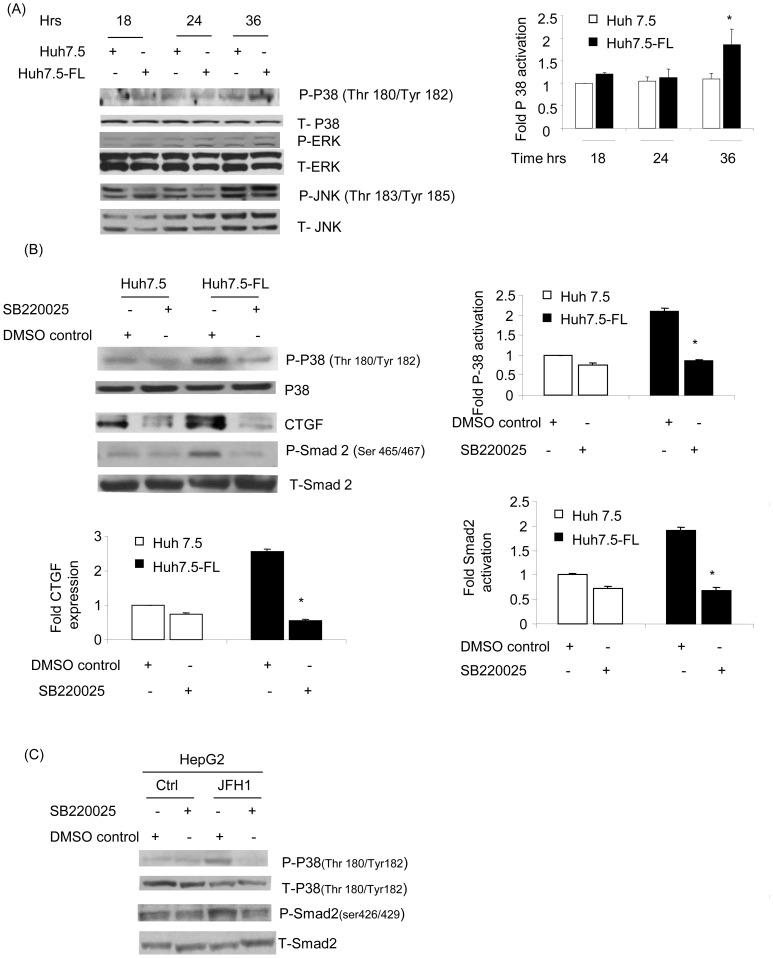
p38 MAP kinase mediates CTGF expression in Huh7.5-FL cells. (**A**) Lysates from Huh7.5 or Huh7.5-FL cells collected at the indicated time points were blotted with Phospho-p38, Phospho-JNK, Phospho-ERK, p38, JNK and ERK antibodies. The bar graph shows the quantitative analysis of p38 activation relative to the total p38 production assessed by densitometry. * P≤0.05 versus Huh7.5 cells. (**B**) Huh7.5 or Huh7.5-FL cells were pretreated with p38 MAPkinase inhibitor (SB220025; 50 µM) for 36 hours, after which cells were lysed and blotted with antibodies to phospho-p38, p38, CTGF, Phospho-Smad2, or Smad2. The bar graph shows the quantitative analysis of the data obtained by densitometry (left panel). * P≤0.05 versus Huh7.5-FL cells. (**C**) Similarly HepG2 cells were transfected with and without JFH1 RNA and cells were treated with p38 MAPKinase inhibitor (SB220025) for 24 hrs ,after which the cell lysates were analyzed for activation of p38 and Smad2. Data represent mean ± SD of 3 independent experiments.

We next investigated the involvement of the major MAPkinase pathways previously implicated in TGF-β1-induced signaling in hepatocytes. We found that p38 MAPkinase was significantly activated in Huh7.5-FL cells in comparison to the Huh7.5 cells, as demonstrated by an increase in phosphorylation at 36 hours (Fig. 7A) of incubation in conditioned medium. There was no major increase in the HCV-induced activation of JNK and ERK 1/2 in Huh7.5-FL cells, as compared to Huh7.5 cells (Fig. 7A). To further investigate the importance of p38 MAPkinase in HCV-induced CTGF production, Huh7.5 or Huh7.5-FL cells were pre-treated with SB220025, a pharmacologic inhibitor of p38 MAPkinase. The reduction in p38 MAPkinase activation (Fig. 7B, first panel) was associated with a concomitant decrease in CTGF protein in conditioned medium (Fig. 7B, third panel). These data clearly suggest the involvement of p38 MAPkinases in HCV-induced CTGF production. Furthermore, we analyzed the cross-talk between p38 MAPkinase and the Smad pathway. We found that Huh7.5-FL cells showed reduced Smad2 phosphorylation in the presence of the specific p38 MAPkinase inhibitor, when compared to control Huh7.5-FL cells (Fig. 7B, panels 4 and 5). To further confirm the role of p38 MAPKinase inhibitor, we used HepG2 cells transfected with control and JFH-1 RNA. As shown in Figure 7C, we also observed significant reduction of p-p38 as well as p-Smad2 in JFH-1 transfected cells. This finding indicates that the phosphorylation of Smad proteins is regulated by p38 MAP kinase. Together, these studies indicate that CTGF production occurs downstream of TGF-β1 and involves a signaling pathway consisting of p38 MAPkinase and Smad group of proteins.

## Discussion

HCV infection is among the leading causes of chronic liver disease. Approximately one third of patients with chronic HCV infection develop significant fibrosis, and many of them develop cirrhosis with a high risk of hepatic decompensation or development of HCC [1]. However, very little is known about the mechanisms by which the virus causes hepatic fibrosis. In this study, we have elucidated for the first time the molecular mechanism of CTGF expression and its role as a mediator of fibrogenesis during HCV infection.

Previously, investigations into the pathogenesis of HCV have been hampered by the lack of *in vitro* and appropriate *in vivo* model systems. However, in the past decade, the establishment of HCV replicons and an infectious cell culture model have allowed for a better understanding of the viral life cycle, pathogenesis of HCV infection and development of antiviral strategies. These two model systems have been widely used to analyze the HCV-mediated mechanisms that lead to liver damage [21], [27]. In the present study, we have shown enhanced expression of CTGF in Huh7.5-FL replicon cells (HCV genotype I) in comparison to Huh7.5 cells. Several previous studies have compared Huh7.5-FL and Huh7.5 cells to study HCV pathogenic mechanisms [28], [29]. In addition, we also used HepG2 cells transfected with JFH1 (HCV genotype 2) to demonstrate increased CTGF expression. Of the six HCV genotypes, viable replicons have been reported for genotype 1 and 2 strains [30]. Hence, we have confirmed increased CTGF expression with both HCV genotypes 1 and 2.

CTGF is a multi-functional protein that drives many cellular processes, but has received special focus with respect to its fibrotic actions in several organs systems. In our study, we have shown that CTGF mediates enhanced expression of fibrotic markers during HCV infection. Specifically, increased expression of several fibrotic markers were observed in Huh7.5-FL cells and CTGF shRNA was effective in reducing procollagen I expression. CTGF produced in response to HCV may act locally on non-parenchymal cells, such as HSCs or myofibroblasts as well as hepatocytes to enhance expression of markers that are associated with fibrosis. Though recent studies have indicated an association between CTGF immunostaining intensity and stage of fibrosis in patients with chronic HCV infection and high levels of CTGF in plasma and liver biopsy samples of HCV infected patients [11], [15], we provide for the first time, clear evidence for the role of CTGF-induced expression of fibrotic markers in HCV infection. Our findings demonstrating increased CTGF expression in HCV-infected hepatocytes also underscore the importance of hepatocytes in producing CTGF during HCV infection. Previous studies have indicated the contribution of parenchymal liver cells to CTGF production in normal and diseased liver [14], [24].

We also investigated the signaling and transcriptional regulatory pathways involved in CTGF expression in HCV-infected hepatocytes. CTGF expression in fibrotic tissue is shown to be either TGF-β1-dependent or independent [10], [24], [31]. Our results show that TGF-β1 upregulates CTGF expression in HCV-infected hepatocytes. The mechanism involved in HCV-induced TGF-β1 production has been well studied. HCV has been shown to regulate TGF-β1 expression by modulating Ca^2+^ signaling and generation of reactive oxygen species (ROS), which acts through p38 MAP kinase, ERK and JNK and NF-k-B signaling pathways to induce TGF-β1 [32], [33]. In the present study, we demonstrate the downstream mediators of TGF-β1 that induce CTGF production. TGF- β1 is known to mediate its functional effects through the Smad group of proteins. We have shown increased phosphorylation of Smad2 in Huh7.5-FL as well as in JFH-1 transfected HepG2 cells compared to control cells. We further demonstrated that TGF-β1-mediated CTGF- production in Huh7.5-FL cells was Smad-dependent as reduced activity was observed in CTGF promoter reporters in which the Smad or BCE sites were mutated. This is in agreement with recent studies which indicate that TGF-β1-driven CTGF gene expression in other cell types is dependent upon a functional Smad element in the CTGF promoter as well as a BCE element which responds indirectly to TGF-β1 [25]. MAPkinases are downstream signaling partners of TGF-β1 and recently MAPK signaling has been shown to directly regulate CTGF expression in fibroblasts [34]. We showed that activation of p38 MAPkinase, but not of JNK kinase or ERK kinase, is important in HCV-induced CTGF production. Previously, p38 MAPkinase was shown to be enhanced in HepG2 cells transfected with HCV core protein [13]. Together, these findings suggest HCV may mediate CTGF production by modulating Smad and p38 MAPkinase dependent pathways.

**Figure 8:**
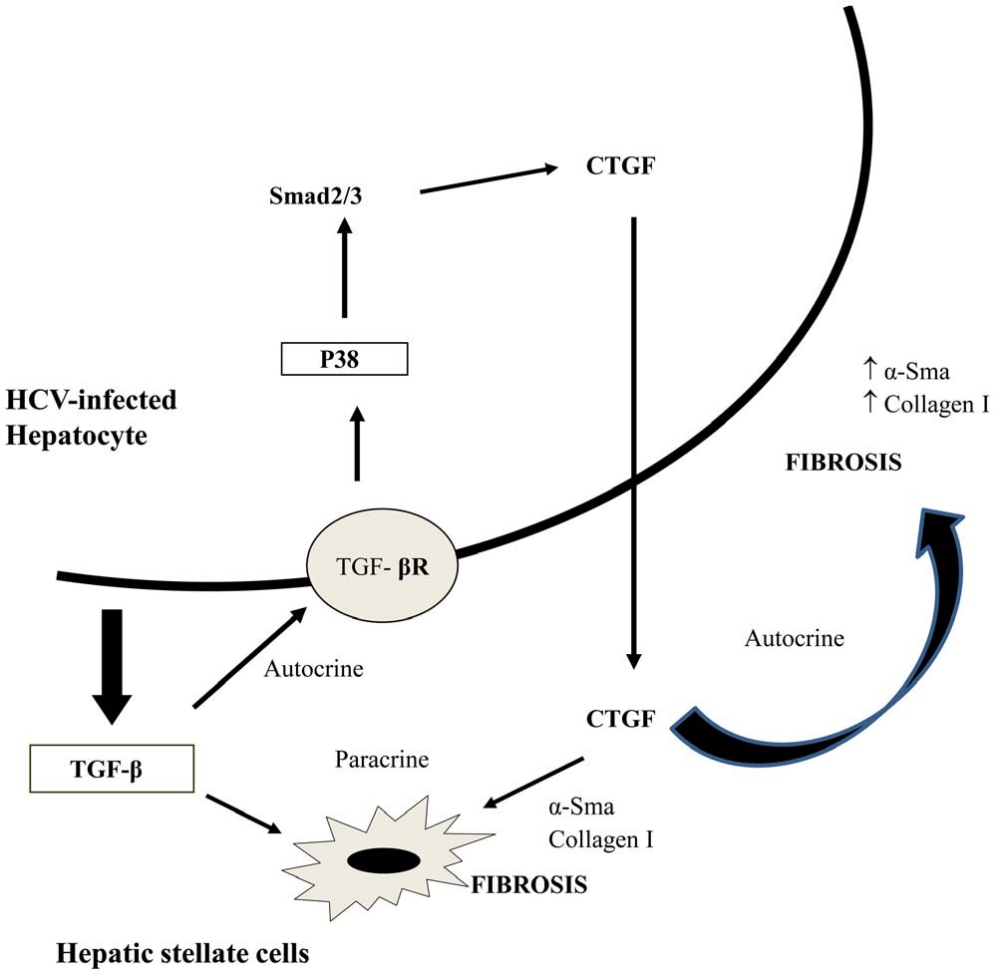
Proposed hypothesis on the role of CTGF in HCV-induced liver fibrosis. We hypothesize that HCV infection in hepatocytes induces TGF-β1 expression. TGF-β1, in turn mediates an enhanced expression of profibrogenic cytokine CTGF through Smad phosphorylation and p38 MAP kinase activation. CTGF may further act in a paracrine manner on hepatic stellate cells (HSCs) or in an autocrine manner on hepatocytes and drive expression of fibrotic markers including collagen and α-Sma.

Based on our studies, we propose a HCV-induced fibrotic pathway in hepatocytes whereby there is an enhanced expression of profibrogenic cytokine CTGF mediated by TGF-β1 through Smad phosphorylation and p38 MAP kinase activation. CTGF, in turn, may act in a paracrine manner on hepatic stellate cells (HSCs) or in an autocrine manner on hepatocytes and drive expression of fibrotic markers including collagen (Fig. 8). Collectively, our data support a role for CTGF as a downstream mediator of the fibrogenic actions of TGF-β1 in promotion of ECM production. The beneficial effect of CTGF knockdown by gene silencing through shRNA has been shown independently in two models of rat liver fibrosis [35], [36]. Our studies underscore the importance of CTGF in HCV-mediated fibrotic pathology and may facilitate the development of anti-fibrotic strategies in chronic-HCV infected patients.
