# Correction: Activation of the Connective Tissue Growth Factor (CTGF)-Transforming Growth Factor β 1 (TGF-β 1) Axis in Hepatitis C Virus-Expressing Hepatocytes

**DOI:** 10.1371/journal.pone.0290786

**Published:** 2023-08-24

**Authors:** Tirumuru Nagaraja, Li Chen, Anuradha Balasubramanian, Jerome E Groopman, Kalpana Ghoshal, Samson T Jacob, Andrew Leask, David R Brigstock, Appakkudal R Anand, Ramesh K Ganju

There is an error in Fig 3A, where the GAPDH results of Figs 1C and 2D were inadvertently included in Fig 3A during figure preparation. The updated Fig 3 provided with this notice shows the correct Fig 3A. The authors explain that the Figs 1C and 2D GAPDH results are identical as the results presented in these figures originate from the same experiment.

**Fig 3:**
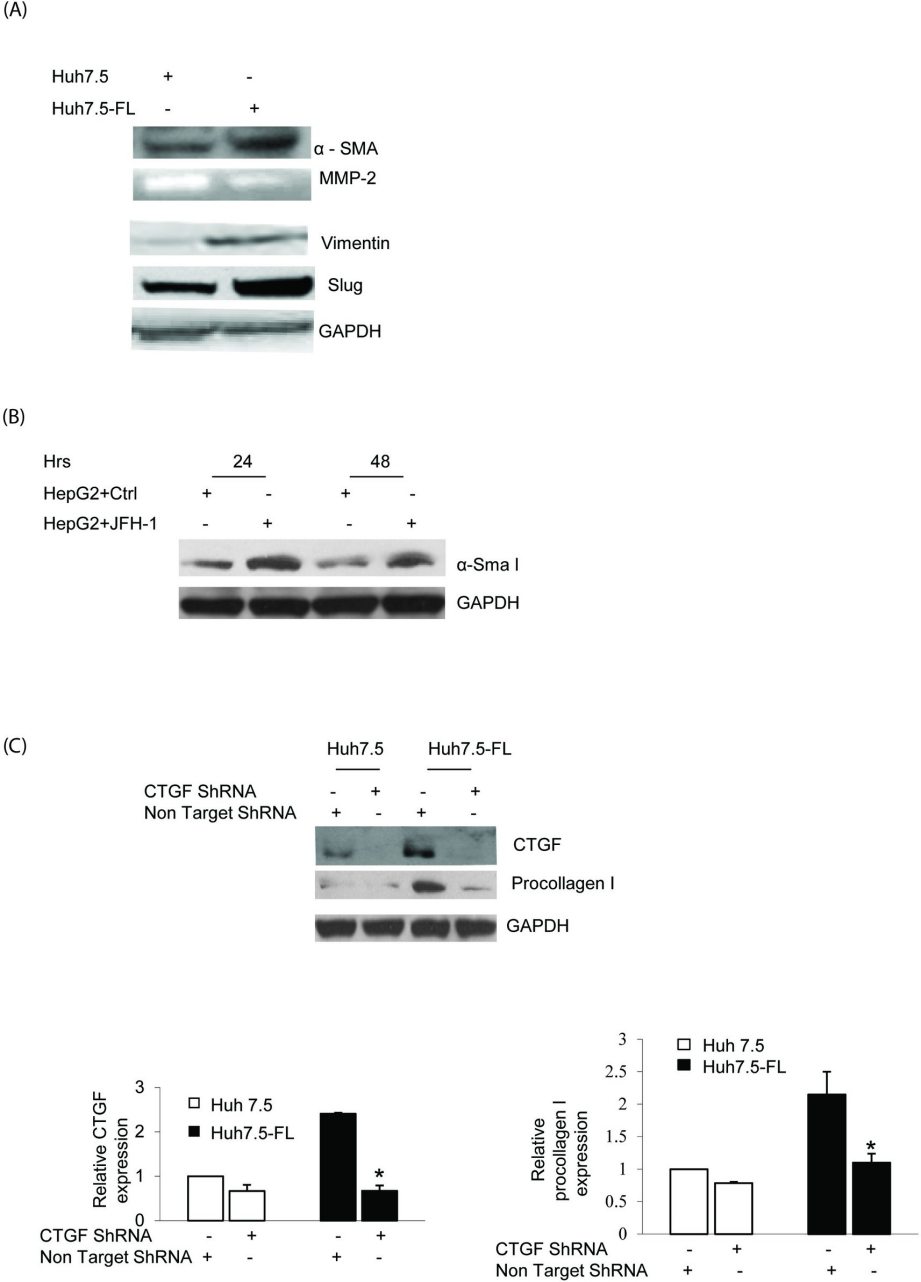
CTGF stimulates the expression of fibrotic markers in Huh7.5-FL cells. (**A**) Huh7.5 or Huh7.5-FL cells were incubated in conditioned medium (medium containing 0.5%FCS) for ninety-six hours and the cell lysates were blotted to examine α-SMA expression, vimentin and slug expression. Equal protein loading was verified using GAPDH antibody. The conditioned medium was used for the measurement of MMP-2 activity by zymography assay. (**B**) HepG2 cells were transfected with or without JFH-1RNA for different time points and cell lysates were blotted for α-Sma I protein. GAPDH was used as an internal control. (**C**) Lysates of Huh7.5 or Huh7.5-FL cells transfected with non targeting or CTGF shRNA for 48 hrs were blotted for CTGF, procollagen I or GAPDH. The bar graphs show the quantitative analysis of CTGF or procollagen I expression relative to that of GAPDH. * P≤0.05 versus Huh7.5-FL cells. Data represent mean ± SD of 3 independent experiments.

The original data underlying the Fig 2A results are provided in S1 File below. The underlying data for all other results presented in this study are no longer available.

## Supporting information

S1 File Original data underlying the Fig 2A results. (XLS)
